# Ventrofixed Uterus: Unfreezing the Uterus in 6 Standardized Steps

**DOI:** 10.1089/gyn.2023.0041

**Published:** 2023-10-03

**Authors:** Tanushree Rao, Sandesh Kade

**Affiliations:** ^1^ Liverpool Hospital, Liverpool, New South Wales, Australia.; ^2^ Sunrise Hospital, Solapur, Maharashtra, India.

## Abstract

**Objective:**

This article presents a 6-step laparoscopic technique for dissecting a central uterine band in a ventrofixed uterus, in order to minimize injury to adjacent structures during such procedures as repeat cesarean sections and hysterectomy.

**Methods:**

The description of this laparoscopic surgical technique shows how the anatomically consistent avascular space beneath the uterine band was accessed via lateral dissection. An online video demonstrating the anatomy, anatomical free space, and secure dissection techniques is included.

**Results:**

The proposed technique enables safe dissection of the uterine band and reduces the risk of bladder injury during uterine-preserving procedures. Accessing the anatomical free space via lateral dissection results in a safer operative field, decreased blood loss, and preserved myometrium during uterine-preserving procedures.

**Conclusions:**

The anatomically consistent avascular space beneath the uterine band is accessible via lateral dissection, enabling secure dissection of the uterine band. This technique can be used in both laparoscopic and open procedures, such as repeat cesarean sections. Familiarity with the anatomy of the central uterine-adhesion band can ensure a safe operation and reduce the risk of bladder injury. (J GYNECOL SURG 39:220)

## Introduction

**FIG. 1.**
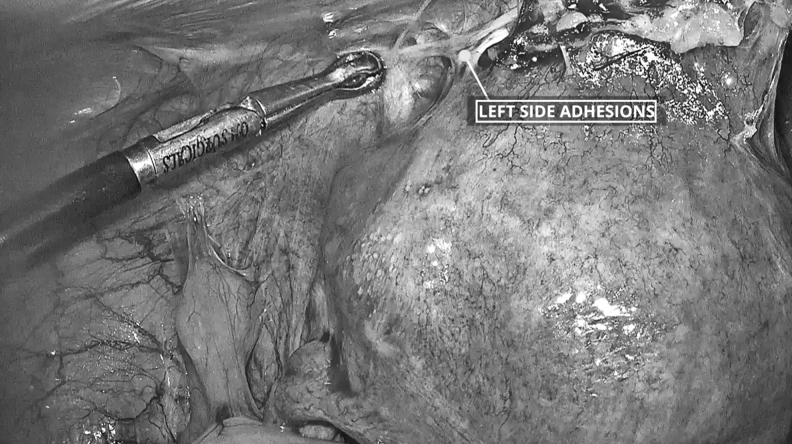
Ventrofixed uterus with uterine adhesions attached to the anterior abdominal wall.

The aim of this article is to show a previously undescribed but anatomically consistent avascular window beneath the uterine band in a ventrofixed uterus as seen in Figure 1 and to demonstrate surgical freeing of central adhesions in 6 standardized and reproducible steps.

**FIG. 2.**
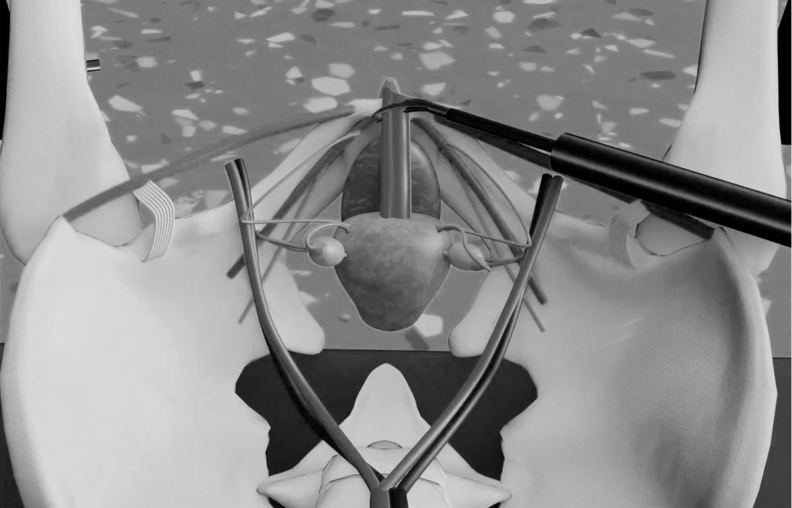
Anatomy of the central uterine adhesion or band and its relationship to surrounding structures.

As the incidence of caesarean procedures increases, so does the prevalence of central uterine bands that ventrofix the uterus.^1^ Sheth described clinical and sonographic signs used to diagnose uterocervical adhesions to the abdominal wall following caesarean section.^2^ However, literature on surgical management of these adhesions is limited. These adhesions, characterized by central uterine bands connecting the uterus to the parietal peritoneum, often arise mid-uterus and include the niche. To perform a hysterectomy, niche repair, and, in some cases, repeat cesarean sections, it is necessary to dissect these adhesions down to access the uterovesical fold. Familiarizing oneself with the anatomy can ensure safe surgery and reduce the risk of bladder injury. A consistent anatomical free space between the bladder and this uterine band can be approached laterally approach, thus isolating the uterine band as seen in Figure 2. An online video (supplementary Video V1; supplementary material is available online at www.liebertonline.com/GYN) highlights the anatomy, the anatomical free space, and ways to dissect it safely.

## Technique

A total laparoscopic approach to dissecting a central uterine band includes the key steps below to minimize injury to surrounding structures:

Step 1—Identify and dissect the left round ligament; dissection should be parallel and above this ligament.
*Step 2*—Identify the left uterine artery.
*Step 3*—Identify and dissect the right round ligament.
*Step 4*—Identify the right uterine artery.
*Step 5*—Identify and dissect the avascular space between the bladder and central uterine band bilaterally.
*Step 6*—Separate the central uterine adhesion band from the anterior abdominal wall (parietal peritoneum).

Note that institutional review board was not required for this project, as confirmed by consultation with Liverpool Hospital, in Liverpool, New South Wales, Australia. This was because the project's design and methodology did not pose any risk to human or animal subjects and complied with ethical research practices.

## Discussion

When dissecting the uterine band during repeat cesarean sections, hysterectomies, or other uterine procedures, one runs the risk of injuring the bladder and triggering excessive bleeding. To minimize the risk of bladder injury during surgery, a cervical cup or uterine manipulator can be used to enhance visualization and precision during dissection. Additionally, ICG dye can be used to clearly delineate the borders of the bladder using near-infrared technology.

## Conclusions

This article brought attention to a potential avascular space/window and how, by utilizing this space, the uterine band can be separated from the underlying lower-uterine segment and bladder, thereby increasing the safety of the operative field, reducing blood loss and conserving myometrium in uterine-preserving procedures. The advantage of detecting and dissecting this window can be utilized in both laparoscopic and open operations, such as repeat caesarean sections, as necessary.

## Supplementary Material

Supplemental data
